# High-rate mechano-stimulation alters proliferation- and maturation-related signaling of oligodendrocyte precursor cells in a 3D hydrogel

**DOI:** 10.1016/j.mbm.2025.100126

**Published:** 2025-03-11

**Authors:** Ryosuke Yokosawa, Rachel A. Mazur, Kelsey A. Wilson, Jacob H. Lee, Noah W. Showalter, Kyle J. Lampe, Pamela J. VandeVord

**Affiliations:** a Department of Biomedical Engineering and Mechanics, Virginia Polytechnic Institute and State University, Blacksburg, VA, 24061, USA; b Department of Chemical Engineering, University of Virginia, Charlottesville, VA, 22903, USA; c Veterans Affairs Medical Center, Salem, VA, 24153-6404, USA

**Keywords:** Traumatic brain injury, Oligodendrocyte precursor cell, Hydrogel, Cell proliferation, Cell maturation, Mechanobiology

## Abstract

Traumatic brain injury (TBI) leads to neuroinflammation and is associated with chronic neurodegeneration. Many TBI studies aim to understand further the mechanism by which cells in the brain respond to the mechanical forces associated with TBI. In particular, mild TBI is the most common level of injury among TBI patients, and the reactivity of glial cells is a key mechanism in understanding mild TBI. However, there is a lack of studies focusing on oligodendrocyte precursor cells (OPCs). OPCs respond to the injury by migration, proliferation, and differentiation into oligodendrocytes (OL) to assist in post-injury repair. Given their ability to proliferate and differentiate, OPCs are a promising therapeutic target for OL regeneration. Despite their important role in maintaining normal neuronal functions, the response of OPCs to mechanical insult remains poorly understood. Thus, this study aims to elucidate the cellular responses of OPCs using a brain-tissue mimicking *in vitro* 3D hydrogel platform to identify key signaling pathways driving their response. In this study, we applied a high-rate pressure wave to OPCs to induce mild TBI and assess subsequent cellular and molecular responses by quantifying cell growth, metabolic activity, and gene and protein expression. Although the high-rate mechanical insult did not significantly impact cell survival, it induced transcriptomic and proteomic changes in molecular targets related to OPC proliferation and maturation, including *PDGFRA*, *GALC*, CTNNB1, and HSP90AB. These dysregulations and altered molecular profiles provide valuable insights into the OPC injury response and may serve as potential therapeutic targets for treating neurodegeneration.

## Graphical abstract

**Figure:**
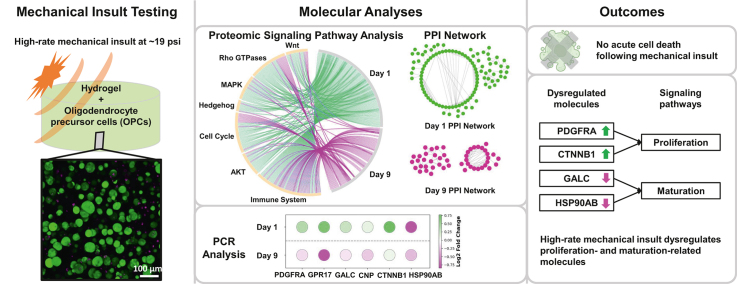

## 1 Introduction

*In vitro* models of traumatic brain injury (TBI) aid in investigating injury mechanisms by studying specific cellular responses to a high-rate mechanical insult. Particularly mild TBI due to the high-rate mechanical insult shows a unique injury mechanism involving glia reactivity without creating visible scars. A widely reported outcome following TBI is neurodegeneration, and neurons are known to be particularly susceptible to mechanical insults.^1^, ^2^, ^3^ However, studies on the cellular responses of glial cells following mechanical insult, without interaction of inflammatory molecules, are limited.^4^, ^5^, ^6^ Neurodegeneration following TBI has been correlated with a loss of myelin and oligodendrocytes (OL) likely caused by secondary injury cascades triggered by the insult.^7^^,^^8^ Research suggests that the proliferation and differentiation of oligodendrocyte progenitor cells (OPC) can be a potential therapeutic target to promote remyelination.^9^, ^10^, ^11^ However, the fundamental cell responses of OPCs following mechanical insult are widely understudied.^7^^,^^12^^,^^13^

**Fig. 1:**
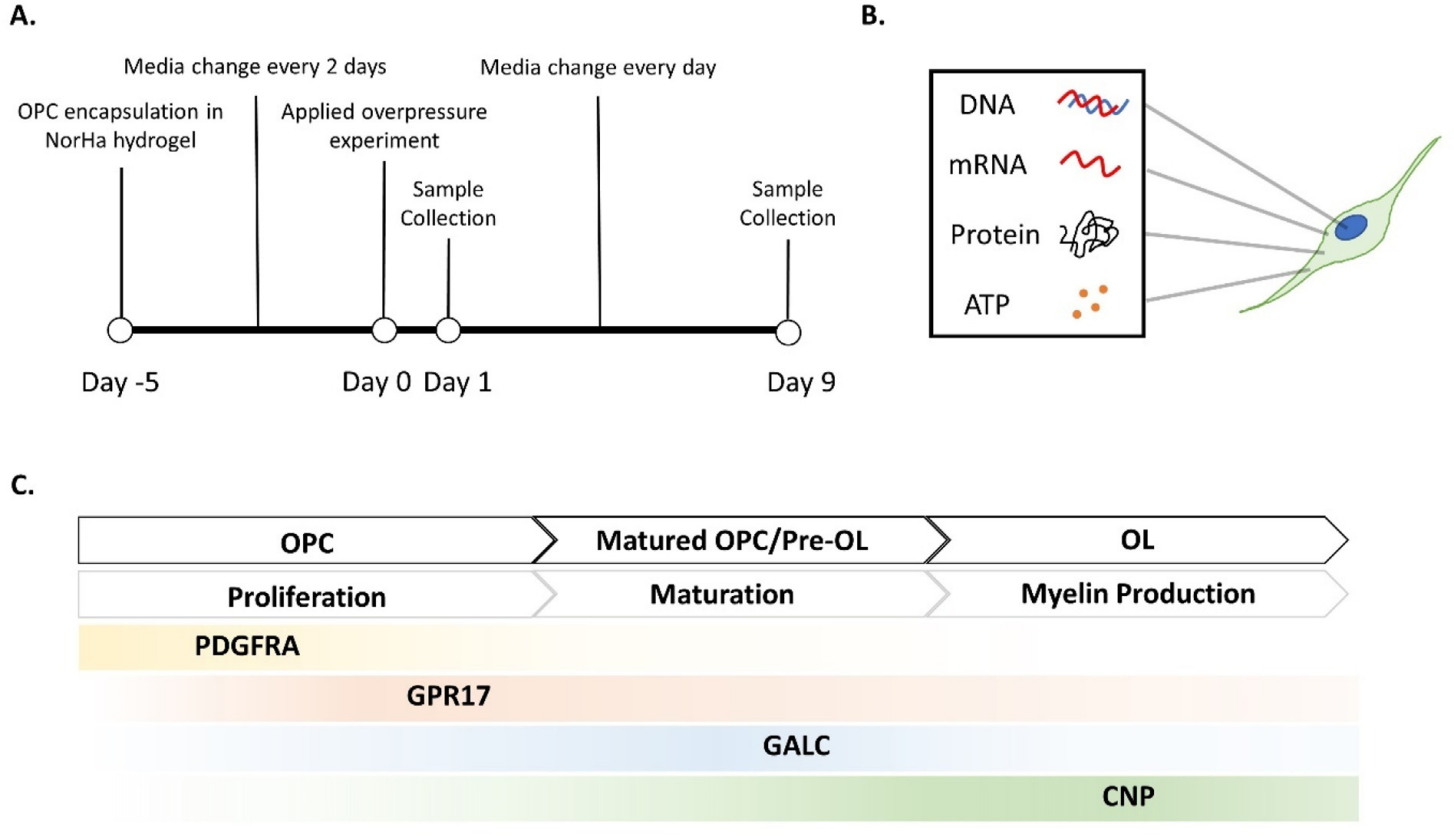
Experiment timeline, collected molecules, and OPC to OL maturation process with targeted molecules. **(A)** Day −5: OPC encapsulation in NorHA hydrogel; Day −4 to −1: Cell culture in OPC growth media and the media was replaced every other day; Day 0: Mechanical insult testing; Day 1: Sample collection; Day 2–8: Continued cell culture in OPC growth media and the media was replaced daily; Day 9: Sample collection. **(B)** Molecular targets to investigate OPC responses after mechanical insult. **(C)** Potential cell maturation process and maturation markers probed for each cell type.

OPCs have three key actions: proliferation, migration and differentiation.^10^^,^^11^^,^^14^ Differentiation of OPCs after migration and proliferation within the injured brain is considered a tissue recovery process. Differentiation into OLs has been investigated and reviewed based on specific protein and gene expressions such as platelet-derived growth factor receptor alpha (*PDGFRA*), G-protein-coupled receptor 17 (*GPR17*), galactosylceramidase (*GALC*), and 2′, 3′-cyclic nucleotide 3′-phosphodiesterase (*CNPase*) (Fig. 1).^13^^,^^15^, ^16^, ^17^ PDGFRA, expressed on the cell membrane, is an early marker of OPCs indicating the ongoing proliferation process.^18^, ^19^, ^20^, ^21^, ^22^, ^23^ GPR17 is a membrane protein whose expression inhibits OPC maturation as the cell enters the maturation phase.^17^^,^^22^, ^23^, ^24^, ^25^, ^26^ GALC, a glycolipid protein, is observed in myelinating OL, whereas the enzyme.^19^^,^^27^, ^28^, ^29^, ^30^, ^31^, ^32^ CNPase and the myelin basic protein (MBP) are fundamental elements of mature OLs when constructing the myelin sheath.^18^^,^^32^, ^33^, ^34^, ^35^, ^36^, ^37^ A recent *in vitro* study demonstrated that abrupt chemical stimulation of the exogenous PDGF-AA, followed by removal of PDGF-AA from culture media, promoted OPC differentiation suggesting its importance in the OPC maturation process.^38^

Innovation in 3D *in vitro* models is an important step in investigating OPC-focused responses to mechanical insult by excluding other glia that are known to initiate inflammatory responses following injury. A hydrogel system provides a more physiologically relevant 3D environment for OPC response than traditional 2D cultures.^39^^,^^40^ This advancement is necessary to replicate the *in vivo* responses more accurately.^41^, ^42^, ^43^, ^44^ One of the advantages of using the hydrogel system is to mimic the storage modulus of native brain tissue with the extracellular matrix (ECM) between 0.2 and 2 ​kPa;^45^ this is vital, as cell responses and reactivities are significantly affected by their environmental cues.^41^ Norbornene-modified hyaluronic acid (NorHA) hydrogels are particularly interesting for neural tissue engineering applications since HA is a key component of brain ECM, particularly within the perineuronal net.^46^ NorHA hydrogel mechanical properties can be easily tuned through thiol-norbornene chemistry and can replicate the stiffnesses of native brain tissue.^45^^,^^47^ Previous studies have introduced the benefits of a NorHA hydrogel system for OPC culture that minimizes hydrogel swelling and maintains high OPC viability for at least seven days.^45^ Cellular activity was reflected by measuring population growth and metabolic activity. Here, assessing cell population growth following mechanical insult is fundamental, providing insights into OPC proliferation post-injury. As demonstrated by Unal et al., the proliferation of cells is quantified by total cell volume and DNA concentration within a hydrogel.^45^ Metabolic dysfunction as a cellular response has been observed following mechanical insult, and previous *in vitro* TBI studies have demonstrated aberrant ATP concentration in astrocytes.^48^^,^^49^ Consequently, implementing the NorHA hydrogel OPC culture system, coupled with metabolic activity and proliferation measures, offers a valuable platform for translational studies of OPC responses to mechanical insults.

The present study exposed OPC-encapsulated NorHA hydrogels to a high-rate pressure wave to investigate their cellular responses and molecular alterations. These results add insight into the critical cellular cues driving OPC proliferation and differentiation post-insult, helping future studies identify potential molecular targets that promote OPC proliferation and, specifically, maturation following TBI.

## 2 Materials and methods

### 2.1 OPC culture

Zong et al. developed the MADM OPC line utilized for these experiments.^50^^,^^51^ A T75 tissue culture flask surface (FB012937, Thermo Fisher Scientific, Waltham, MA) was treated with 10 ​μg/mL polyornithine (P3655, Sigma–Aldrich, St. Louis, MO) then rinsed three times with Dulbecco's phosphate buffered saline (DPBS, 14190250, Life Technologies, Carlsbad, CA). OPCs were cultured in growth media: Dulbecco's modified Eagle's medium (DMEM) with 4.5 ​mg/L d-Glucose and 110 ​mg/L sodium pyruvate (11995073, Life Technologies, Carlsbad, CA) supplemented with N2 supplement (17502048, Life Technologies, Carlsbad, CA), B27 supplement (17504044, Life Technologies, Carlsbad, CA), and 1 ​% penicillin-streptomycin (P4333, Sigma–Aldrich Inc., St. Louis, MO). Frozen OPCs (1 ​× ​10^6^ cells per vial, passages 20–30) were thawed in a water bath at 37 ͦC and seeded at 1.0 ​× ​10^5^ cells/mL in growth media. Cells were incubated at 37 ͦC and 5 ​% CO_2_. The media was changed every two days until the confluency reached 80 ​%.

### 2.2 OPC encapsulation in hydrogels

NorHA hydrogel material was synthesized and characterized following Unal et al.*’s* previously reported protocol.^45^ Before use, lyophilized NorHA was sterilized with UV irradiation for 20 ​min in a biosafety cabinet. All materials were solubilized and diluted in DPBS as a stock solution prior to the formulation of the hydrogel. A 1.5 ​% NorHA concentration was chosen to replicate brain tissue stiffness based on the previously characterized storage modulus (793.9 ​± ​203.3 ​Pa).^45^ The hydrogel precursor solution was prepared to a final concentration of 1.5 ​% NorHA by combining 9 ​mg/mL NorHA, 6 ​mg/mL of dithiothreitol (DTT, D0632, Sigma–Aldrich Inc., St. Louis, MO), and 23 ​mM lithium phenyl-2,4,6-trimethylbenzoylphosphonate photoinitiator (LAP, 900889, Sigma–Aldrich Inc., St. Louis, MO). The cell suspension was combined with the premixed solution at a final concentration of 5 ​× ​10^6^ cells/mL. A tip was removed from the 1 ​mL BD Disposable Syringes with Luer-Lok™ Tip (309628, BD, Franklin Lakes, NJ), and the syringe was used as hydrogel molds. 40 ​μL of the hydrogel solution (200,000 cells per gel) was added to the syringe. The syringe was vertically placed under the UV chamber and then exposed to 365 ​nm UV light at 4 ​mW/cm^2^ for 2 ​min. OPC-encapsulated hydrogels were collected from the syringes and rinsed three times in DPBS. Hydrogels were incubated in the OPC growth media for 5 days (Day −5 to 0 in Fig. 1A), replacing the media every other day until the day of the mechanical insult testing.

### 2.3 Mechanical insult testing

**Fig. 2:**
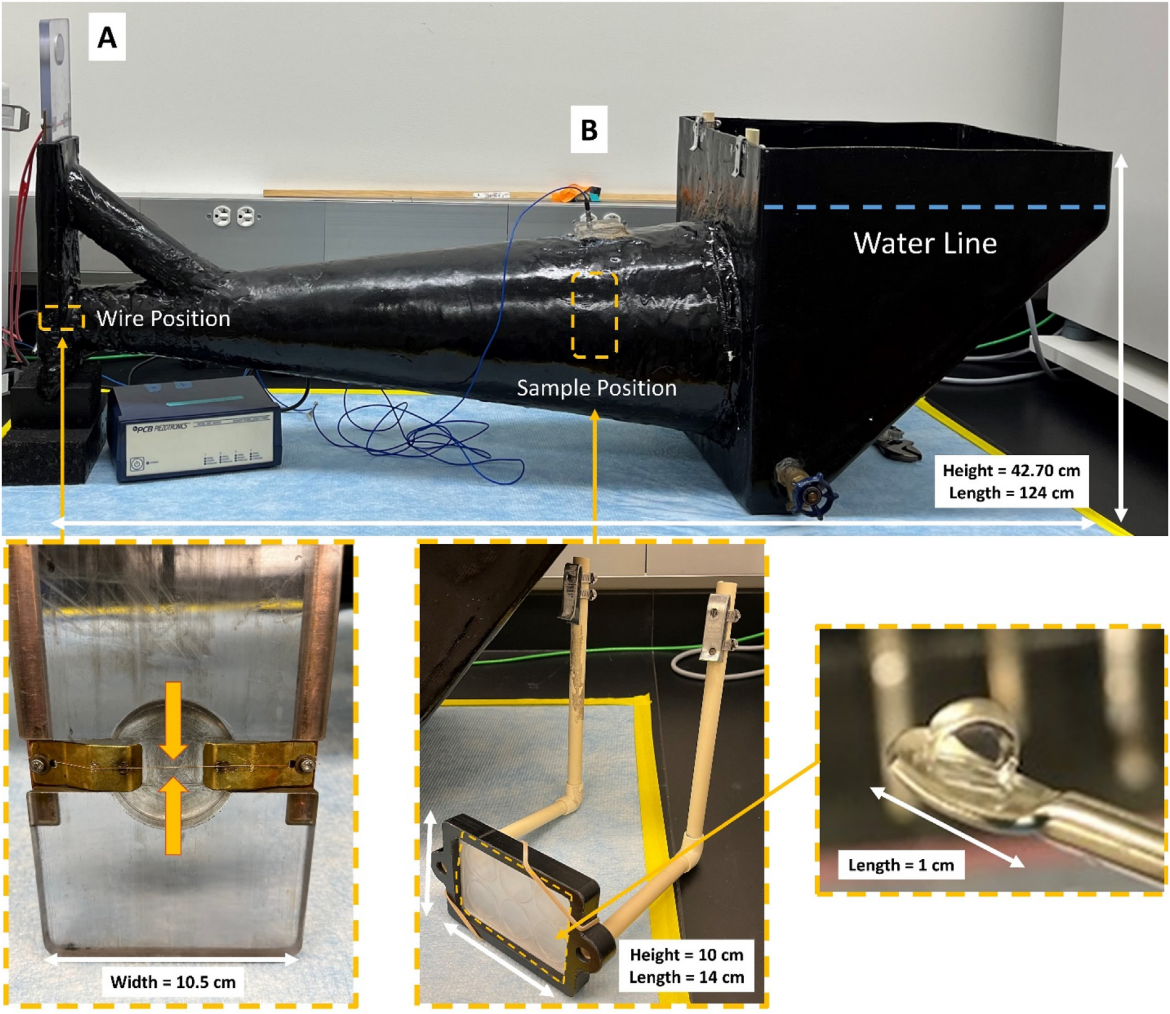
Shock Wave Generator (SWG) system for *in vitro* mechanical insult testing. **(A)** Bridging-wire detonation board with loaded thin metal wire. A thin metal wire was fixed on the board (between two arrows). **(B)** A pressure sensor was mounted above the samples' location (dashed line square). The sensor was placed flush on the inner surface of the SWG chamber, orthogonal to the well plate. The water line was annotated with the blue dashed line. OPC encapsulated hydrogel (bottom right magnified picture) was placed in a culture plate sealed with parafilm and then tightly fixed to the plate holder with a rubber band on the corners to maintain plate location throughout mechanical insult testing. White arrow indicates the length of each object.

As characterized in previous studies, a shock wave generator (SWG) was used to subject OPCs (37 ͦC) to a high-rate pressure wave as a mechanical insult.^52^^,^^53^ This device was developed to mimic intracranial pressures found within the rodent brain during a TBI insult, which also presents with neuropathology (Fig. 2).^54^, ^55^, ^56^, ^57^ The SWG operates on a bridging-wire detonation system generating a high-rate mechanical insult. By applying a high voltage through a nichrome wire (0.003 inches diameter, Part# 40BNC, Consolidated Electronic Wire & Cable, Franklin Park, IL), a single high-rate pressure wave was generated (Fig. 2A). The applied high-rate pressure waves were measured by a piezoelectric pressure sensor (Model 113B21, PCB Piezotronics Inc., Depew, NY) (Fig. 2B).

After five days of cell culture, OPC-encapsulated hydrogels were exposed to the applied insult on Day 0. Two gels were placed in each well of a 6-well cell culture plate (08-772-3A, Thermo Fisher Scientific, Waltham, MA), totaling 12 gels per plate. DMEM without supplements was added to fill the plate, and parafilm was tightly sealed over the plate to exclude bubbles and allow the transmission of the high-rate pressure wave propagation to the cells. The test plate was tightly attached to the plate holder (Fig. 2C) and submerged in the SWG chamber. OPC-encapsulated hydrogels were exposed to a single insult lasting milliseconds. The OPC-encapsulated hydrogels were then retrieved from each well and transferred to a 24-well plate (08-772-1, Thermo Fisher Scientific, Waltham, MA) with 1 ​mL of growth media per well. Sham samples were prepared by following the same steps without exposure to a mechanical insult. OPCs in hydrogels were cultured for one or nine days after the exposure. The media was replaced every day until the day of sample collection.

Mechanical exposure data were collected at 1 ​MHz on a Dash 8HF-HS system (Astro-Med, Inc., West Warwick, RI) with a 4-channel signal conditioner (Model 482C05, PCB Piezotronics Inc., Depew, NY). Acquired data were analyzed and visualized in MATLAB (R2021b, The MathWorks Inc., Natick, MA), where average peak pressure, rise time, positive duration, and positive impulse were reported.

### 2.4 NorHA hydrogel characterization: oscillatory shear rheology

After encapsulating OPCs in 1, 1.5, and 2 ​wt% NorHA hydrogels, OPCs were incubated at 37 ​°C for five days and subjected to applied insult or sham conditions. We used the range of 1–2 ​wt% NorHA hydrogels to identify suitable hydrogel conditions for additional studies. The material properties of each hydrogel were then assessed 8–12 ​h after insult. OPC-encapsulated hydrogels were placed onto an Anton Paar MCR 302 rheometer (Anton Paar, Torrance, CA), with an 8 ​mm diameter parallel plate insert. Oscillatory frequency sweeps were conducted with a gap height of 1.6 ​mm, oscillatory strain of 0.1 ​%, and an angular frequency ranging from 0.1 to 10 ​rad/s. As a result, 1.5 ​wt% NorHA gel best met our criteria of mimicking brain tissue while also providing suitable mechanical properties for manipulation and cell culture. Since this particular condition was identified as a suitable culture system, all further cell culture was conducted in 1.5 ​wt% NorHA hydrogels.

### 2.5 Cell viability assay

OPC viability in NorHA hydrogels was quantified using the LIVE/DEAD™ Viability/Cytotoxicity Kit (L3224, Thermo Fisher Scientific, Waltham, MA) on either Day 1 or 9 post-insult. Briefly, hydrogels were washed with DPBS and placed in 1 ​mL of DMEM lacking phenol red (31053028, Thermo Fisher Scientific, Waltham, MA). Live cells were imaged for GFP expression on the MADM OPCs, and dead cells were stained with a 4 ​μM ethidium homodimer 1 (EthD1) with incubation at 37 ​°C for 30 ​min, followed by rinsing twice with DPBS. Live/Dead images were captured using a Zeiss LSM800 MA-Pmt2 confocal microscope with 300–600 ​μm z-stacks at 5 ​μm slices using the 10x objectives. Three hydrogel samples per condition per experiment were collected. Three regions of interest (ROI) were selected for each gel between the center and edge of the hydrogel. Images were analyzed in ImageJ using the 3D Object Counter plugin.

### 2.6 Metabolic activity assay

Cytosolic adenosine triphosphate (ATP) activity in OPCs was quantified using the CellTiter-Glo® 2.0 Cell Viability Assay (G9241, Promega Corporation, Madison, WI). Hydrogel samples (n ​= ​3 per condition per experiment) were collected in 400 ​μL of Passive Lysis 1X Buffer (E1941, Promega Corporation, Madison, WI) diluted in DPBS, homogenized using a disposable pellet mixer (47747-358, VWR, Radnor, PA), and sonicated three times at 40 ​J for 10 ​s. ATP standards were prepared following the manufacturer protocol, and samples were diluted 1:4 in DPBS. Each sample was run in triplicate with luminescence measured by the SpectraMax M2e microplate reader (Molecular Devices, LLC., San Jose, CA). ATP concentration [ng/mL] of each sample was then determined.

### 2.7 Double-stranded DNA quantification

Double-stranded DNA (dsDNA) was measured using a fluorescent nucleic acid stain via the Quant-iT™ PicoGreen™ dsDNA Assay Kit (P7589, Thermo Fisher Scientific Inc., Waltham, MA). Hydrogel samples (n ​= ​3 per condition per experiment) were homogenized, as described previously and measured at a 1:4 dilution in triplicate. Standards were prepared following the manufacturer's recommendations, and the plate was allowed to incubate for 10 ​min at room temperature in the dark. The absorbance at 525 ​nm was measured using the SpectraMax M2e microplate reader, and DNA concentrations [ng/mL] were determined.

### 2.8 Spheroid analysis

Spheroid volumes of OPC in hydrogel were quantified using the 3D Object Counter plugin in ImageJ on the Live/Dead confocal images. Live (GFP) and Dead (EthD1) cell images were separately analyzed. The 3D Object Counter computed each spheroid volume by connecting the voxels (64.5 ​nm ​× ​64.5 ​nm X 200 ​nm per voxel) within one spheroid, and the number of spheroids in each hydrogel ROI was counted. Total spheroid volume was calculated by adding the volume of all spheroids per ROI; then three ROI data were averaged to estimate the total OPC volume per ROI in each hydrogel. Mean spheroid volume was calculated by averaging the volume of all spheroids in each ROI, and then the mean spheroid volumes of three ROIs per hydrogel were averaged. Each ROI's total and mean spheroid volumes were calculated for overpressure and sham conditions at each time point. In addition, the occupation ratio of the spheroid within each ROI of the hydrogel was analyzed by normalizing the total spheroid volume to the hydrogel volume at each ROI (638.9 ​μm ​× ​638.9 ​μm x z stack slices x 5 ​μm). The minimum number of voxels was limited to 20 to exclude detected fragments below the single cell volume for all analyses. Three confocal image stacks were taken per sample. Three samples for each test and sham conditions were analyzed from each time point. Four experiments were conducted for 12 samples per condition per time point.

### 2.9 RNA and total protein extraction

Samples were collected on Day 1 and 9 following mechanical insult and stored in 600 ​μL of Trizol reagent (15596018, Thermo Fisher Scientific, Waltham, MA) at −80 ͦC prior to downstream analysis. Samples were homogenized, as described above, and RNA was extracted using the PureLink™ RNA Mini Kit (12-183-018A, Thermo Fisher Scientific, Waltham, MA) following the manufacturer's instruction. RNA purity and integrity were then confirmed using NanoDrop™ One/OneC Microvolume UV-Vis Spectrophotometer (Thermo Fisher Scientific, Waltham, MA); all samples had an A260/A280 nm purity ratio between 1.8 and 2.0. For Western blot analysis, OPC hydrogels (5 gels per vial) were homogenized and protein extracted using the Mem-PER™ Plus Membrane Protein Extraction Kit (89842, Thermo Fisher Scientific, Waltham, MA) following the manufacturer's protocol and subsequently stored at −80 ͦC. The protein concentration of each sample was determined using Pierce™ BCA Protein Assay Kit (23225, Thermo Fisher Scientific, Waltham, MA) following the manufacturer's protocol, and the absorbance at 562 ​nm was measured using the SpectraMax M2e microplate reader.

### 2.10 Reverse transcription quantitative real-time PCR (qPCR)

Changes in gene expression of OPC maturation markers and two selected markers from proteomic analysis were analyzed using qPCR on Days 1 and 9. Briefly, 100 ​ng RNA was converted to cDNA using the iScript™ cDNA Synthesis Kit (1708840, BIO-RAD, Hercules, CA) following the manufacturer's protocol. cDNA was then combined with respective primers and TaqMan™ Fast Advanced Master Mix (4444557, Thermo Fisher Scientific, Waltham, MA), loaded into a 96-well plate with 60 ​ng cDNA per well, and ran in triplicate using the QuantStudio™ 3 Real-Time PCR System (Thermo Fisher Scientific, Waltham, MA). Taqman primers for OPC maturation markers and reference genes were selected as follows: *GAPDH* (Mm99999915_g1, Thermo Fisher Scientific, Waltham, MA), *PDGFRA* (Mm00440701_m1, Thermo Fisher Scientific, Waltham, MA), *GPR17* (Mm02619401_s1, Thermo Fisher Scientific, Waltham, MA), *GALC* (Mm00484646_m1, Thermo Fisher Scientific, Waltham, MA), and *CNP* (Mm01306641_m1, Thermo Fisher Scientific, Waltham, MA). Primers for two selected targets by proteomic analysis (*CTNNB1* and *HSP9*0AB*)* were selected as follows: *CTNNB1* (Mm 00483039_m1, Thermo Fisher Scientific, Waltham, MA) and *HSP9*0AB (Mm 00833431_g1, Thermo Fisher Scientific, Waltham, MA). For qPCR analysis, the delta–delta Ct method (2^−ΔΔCT^) was used to calculate fold changes in samples normalized to *GAPDH* and the respective sham. 3 hydrogel samples per condition per experiment were collected for the analysis.

### 2.11 Western blot analysis

To further investigate if changes were occurring to the proteomic landscape of OPCs when subjected to mechanical insult, Western blot analysis of OPC maturation markers was conducted on Days 1 and 9 using the Jess™ (ProteinSimple, San Jose, CA) combined with the 12–230 ​kDa Separation Module (SM-W001, Bio-Techne, Minneapolis, MN). Protein was extracted and measured following the procedures outlined above. Protein samples (n ​= ​3 per condition per experiment) were diluted to a final concentration of 0.26 ​mg/mL and incubated with their respective antibody diluted 1:250 for chemiluminescent detection. Antibodies used include rabbit anti-PDGFRA (PA5-16571, Thermo Fisher Scientific, Waltham, MA), rabbit anti-GPR17 (PA5-120874, Thermo Fisher Scientific, Waltham, MA), rabbit anti-GALC (11991-1AP, Thermo Fisher Scientific, Waltham, MA), and rabbit anti-CNPase (PA1-46433, Thermo Fisher Scientific, Waltham, MA). Chemiluminescent reactions were recorded through electropherograms, and digital immunoblots were constructed using the Compass SW software (ProteinSimple, San Jose, CA). The area under the electropherogram was normalized to the respective sham control and used to calculate protein expression levels.

### 2.12 Proteomic data acquisition and analysis

Protein samples (100 ​μg, n ​= ​3 samples per condition) were prepared for proteomic analysis at the Virginia Tech Mass Spectrometry Incubator. Briefly, proteins were processed through SDS-PAGE, extracted, reduced with DTT, alkylated, digested with trypsin, and resuspended in 0.1 ​% formic acid as described by Browning et al.^58^ Each sample underwent three injections (1 ​mg each) for Liquid Chromatography MS/MS analysis. Data-independent acquisition (DIA) data analysis was automated using Protalizer DIA software pipeline version 2.1 (Vulcan Analytical, Birmingham, AL), which combined the results from the three injections per sample. Peptides and source proteins were identified through an X! Tandem Sledgehammer MS/MS database search, using the mouse Swiss-Prot reference proteome, followed by deconvolution of MS/MS spectra.

Differentially expressed proteins (DEPs) in OPCs were normalized to their respective controls (day of protein collection, 1 or 9). DEPs were identified using a false detection rate (FDR) ​≤ ​0.05, a peptide spectral match (PSM) ​≥ ​20, and several unique proteins ≥1. By applying the additional cut-off criteria of log_2_ fold change ≥ |1.2| and an adjusted p-value ≤0.1, we identified 217 DEPs in the Day 1 group and 158 DEPs in the Day 9 group. To elucidate the distinctive and temporally dynamic proteomic profiles of OPCs subjected to mechanical insult, functional enrichment analysis of DEPs using Gene Ontology (GO), Kyoto Encyclopedia of Genes and Genomes (KEGG), and Reactome tools through Cytoscape 3.10.1 (Cytoscape Consortium) was performed. The significantly enriched signaling pathways were parsed through PubMed to gain insights into their role in OPC proliferation and differentiation.

To identify the proteins that were the most robust molecular targets predicative of OPCs following applied overpressure on Day 1 and 9, protein–protein interaction (PPI) networks were generated and analyzed using Cytoscape 3.10.1 with the centrality metrics of degree, betweenness, stress, closeness, and eccentricity. Using weights as subjective multipliers for Degree∗0.3, Betweenness∗0.3, Stress∗0.4, Closeness∗0.2, and Eccentricity∗0.1, centrality measurements were manually weighted to determine the most influential proteins from each experimental group. Degree, betweenness, and stress indicated the number of connections between proteins; thus, higher weights were applied, while closeness and eccentricity represented the distance between proteins; lower weights were applied. PageRank was used as a second weighting method in which the proteins were weighted based on their source and target node previously acquired from Cytoscape 3.10.1. The top 10 DEPs from these two ranking methods were then highlighted as the most influential proteins in their respective overall network. Proteins identified to overlap between the two ranking methods were high-confidence molecular targets following mechanical insult.

### 2.13 Statistical analysis

Statistical comparisons were conducted to compare each experimental condition to control using GraphPad Prism 9 (GraphPad Software, San Diego, CA, United States). Outliers were determined by the ROUT method (Q ​= ​1 ​%) and excluded before proceeding to any statistical analysis. The Shapiro–Wilk test with an alpha value of 0.05 verified assumptions of normality. An unpaired t-test was used for all normally distributed data to compare two experimental groups. The Mann–Whitney U test was used when the data was not normally distributed. Differences were considered statistically significant when p ​< ​0.05.

## 3 Results

### 3.1 Characterization of applied mechanical insult

**Fig. 3:**
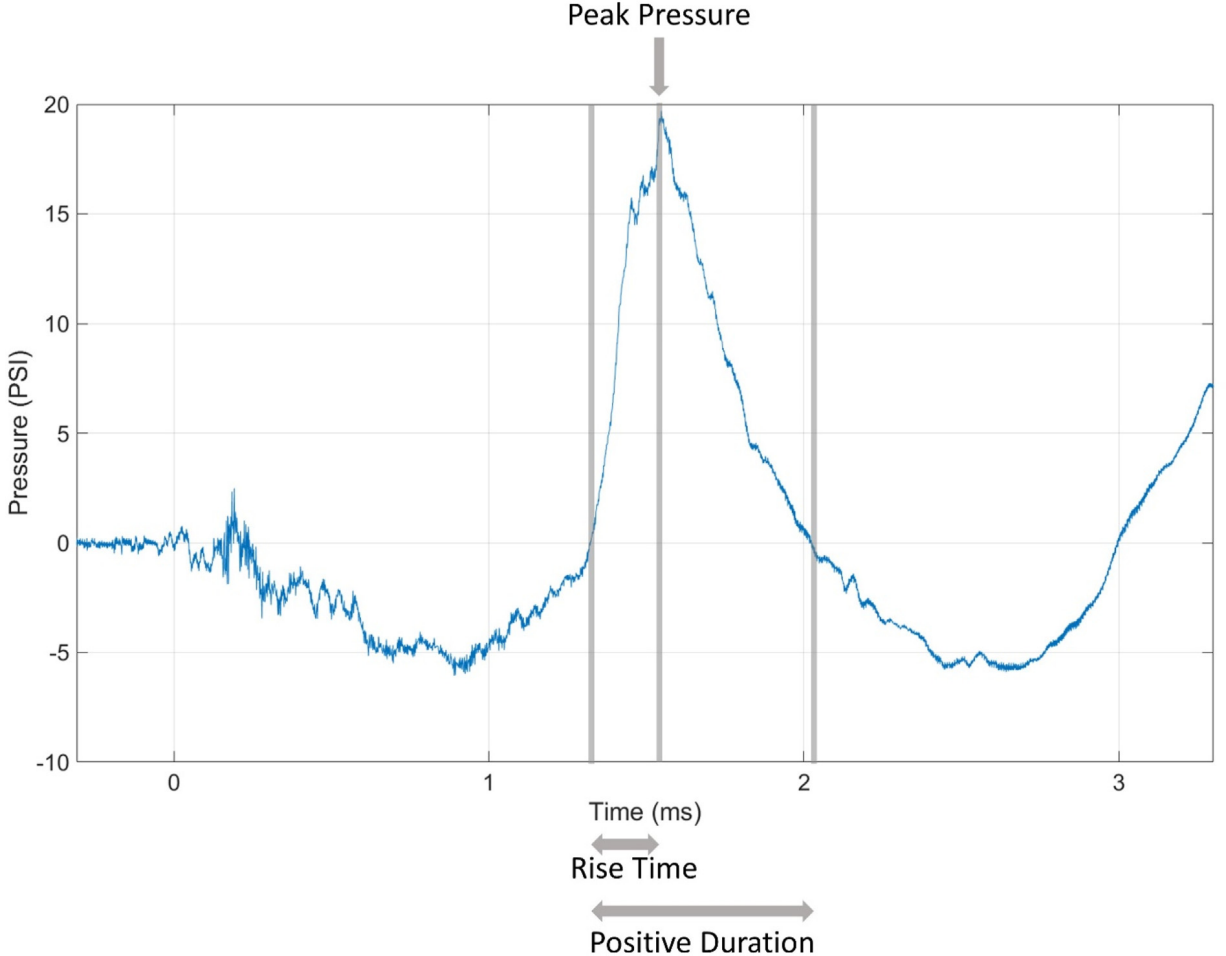
Representative data of a high-rate overpressure profile for a simulated blast. The SWG generated pressure profile is depicted, and annotations for characterized parameters are shown. Grey vertical lines indicate the analyzed range of the overpressure curve. Peak pressure was determined as the maximum of the first peak (rise above 0 PSI) within the range of the drastic pressure rise. Positive impulse was calculated from an integration of pressure within a positive duration. Pressure is in pounds per square inch [psi], and time is in milliseconds [ms] (n ​= ​14).

The mechanical insult parameters generated by the SWG were summarized in Table 1, which characterized the peak pressure, rise time, positive duration, and positive impulse (Fig. 3). Samples were exposed to average peak pressures of 19.08 ​± ​6.53 psi. A total of 14 mechanical insult tests were conducted to collect samples required for multiple molecular analyses in this study.

**Table 1:** High-rate overpressure loading conditions.

Parameter	Average	Standard Deviation
***Peak pressure [psi]***	19.08	6.53
***Rise time [ms]***	0.54	0.47
***Positive duration [ms]***	1.09	0.35
***Positive impulse [psi∗ms]***	5.11	2.06

### 3.2 Characterization of NorHA hydrogel

**Fig. 4:**
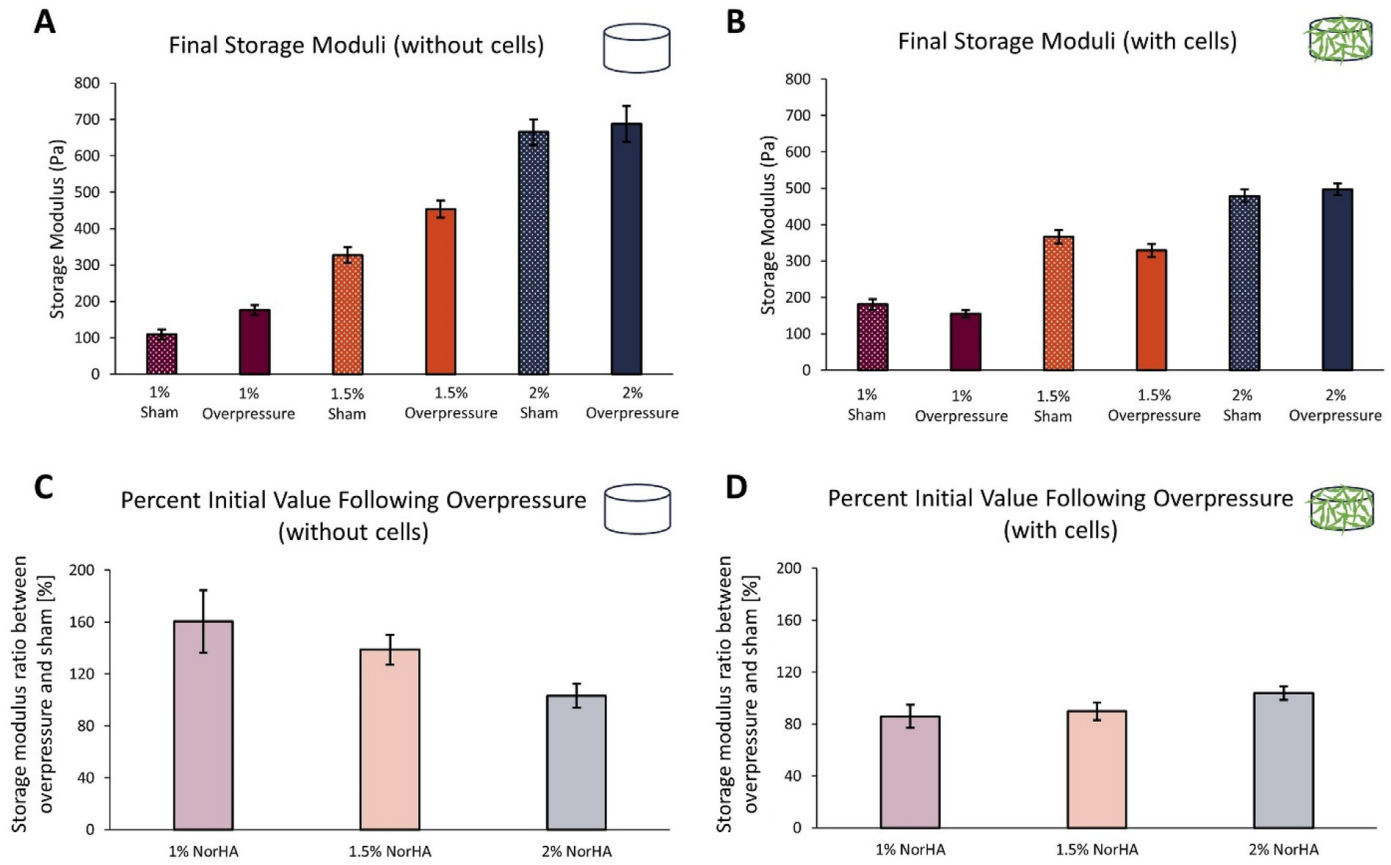
Material properties of NorHA hydrogel with or without cells following mechanical insult. **(A)** Final storage moduli readings for 1–2 ​wt% NorHA gels. (n ​= ​2 samples with no cells) **(B)** Final storage moduli readings for OPC-containing 1–2 ​wt% NorHA gels with an initial cell density of 200,000 cells per gel (5 ​× ​10^6 cells/mL). (n ​= ​2 samples with cells) **(C)** Comparison of acellular NorHA gel stiffness between overpressure and sham conditions. **(D)** Comparison of OPC-containing NorHA gel stiffness between overpressure and sham conditions. Cell-containing gels were cultured to a timepoint of five days and exposed to either overpressure or sham injury conditions. Frequency sweeps were performed with angular frequency between 0.1 and 10 ​rad/s (n ​= ​2 samples). The final storage modulus for each gel was averaged across both gel replicates, and the percent initial value was then calculated using the formula (overpressure/sham) ∗ 100.

Oscillatory shear rheology was performed on 1–2 ​wt% NorHA gels to determine the impact of applied mechanical insult on gel mechanical properties (Fig. 4A and B). Frequency sweeps were conducted on overpressure and sham samples at the three NorHA concentrations. Gel stiffness increased as NorHA concentration increased (Fig. 4A). Analogous experiments were conducted on 1–2 ​wt% NorHA gels containing encapsulated OPCs (Fig. 4B and D). Gels without cells at all NorHA concentrations were found to have higher storage moduli than the corresponding cell-containing gels (Fig. 4A and C). The effect of overpressure on gel stiffness was muted in the presence of cells.

### 3.3 Live/dead spheroid analysis

**Fig. 5:**
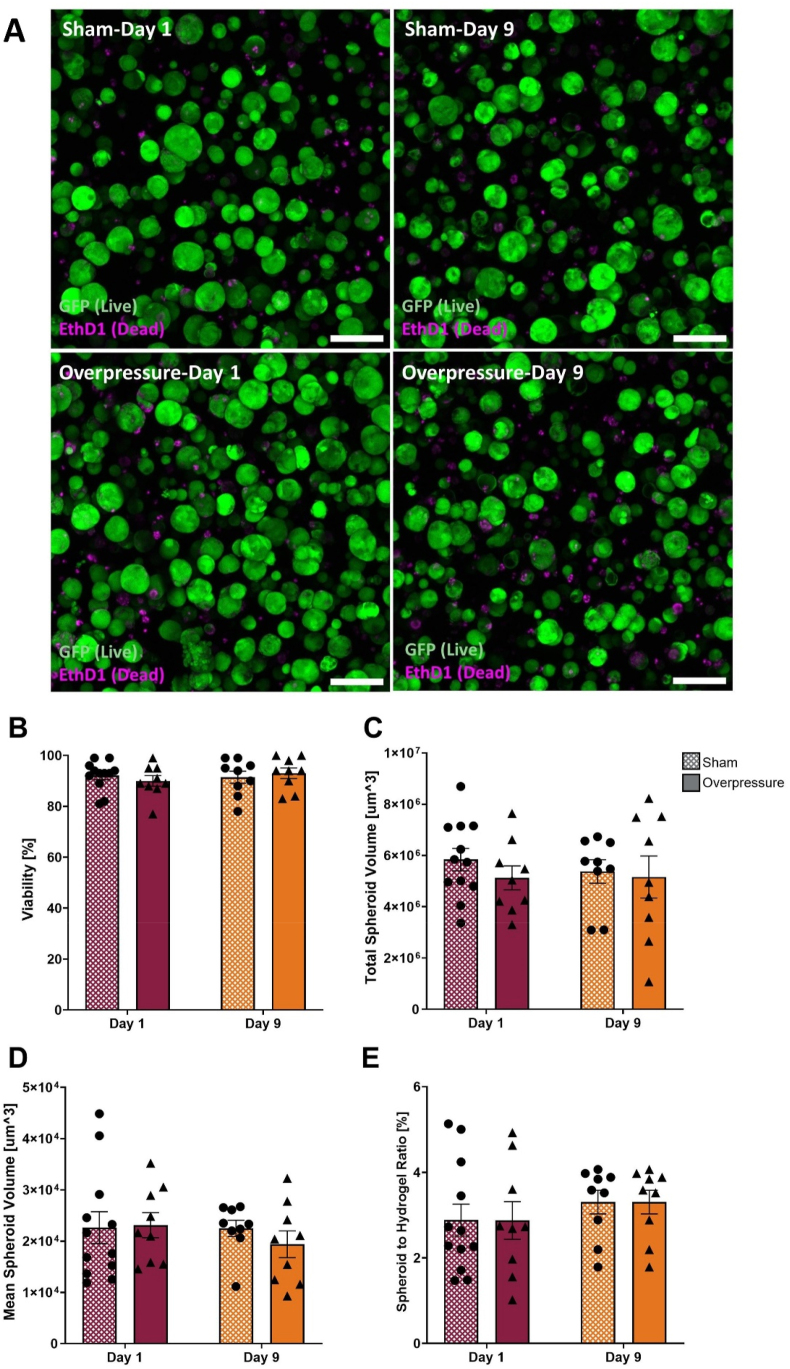
Spheroid analysis was conducted to compare all experimental conditions. **(A)** Representative confocal images of Live/Dead stained spheroids. Live cells are green and dead cells are magenta. Scale bar ​= ​100 ​μm. **(B)** Viability of spheroids per hydrogel, which represents a ratio of the live (green) spheroid total volume to spheroid total volume of live (green), and dead (magenta) spheroids. **(C)** Mean spheroid volume per hydrogel. **(D)** Total spheroid volume per hydrogel. **(E)** Spheroids to hydrogel occupation ratio. Statistical analysis comparing sham and overpressure at each time point showed no significant difference for all data. The unpaired t-test was used for normally distributed data, and the Mann–Whitney U test was used for not normally distributed data. Data represented as Mean ​± ​SEM (n ​= ​9–12 samples).

Live/Dead viability assays were conducted to determine if the applied mechanical insult induced cell death (Fig. 5). Between 72 and 449 spheroids were measured at each ROI for overpressure and sham conditions at each time point (Fig. 5A). The viability of spheroids for all experimental conditions remained in the range of 91.67 ​± ​6.14 ​% (Fig. 5B). Spheroid total volume was not significantly altered between overpressure and sham experimental groups or between time points (Fig. 5C). For every condition, the total spheroid volume of OPC was in the range between 5.12 ​× ​10^6^ and 5.84 ​× ​10^6^ um^3^ except the Day 9 post-overpressure samples which showed higher deviation (5.16 ​× ​10^6^ ​± ​2.46 ​× ​10^6^ um^3^) as compared to other three conditions. On Day 1 following the insult, between sham and overpressure samples, the total volume result showed a 12 ​% decrease in overpressure compared to sham (Fig. 5C). The mean volume of a single spheroid within a hydrogel was measured to quantify the size of each spheroid in a hydrogel. Mean volume showed no significant difference between experimental conditions. However, the mean volume on Day 9 post-insult showed a 16 ​% decrease compared to the sham (Fig. 5D). In addition, the occupation ratio of spheroid volume to hydrogel volume showed between 2.87 ​% and 3.30 ​% in all experimental conditions (Fig. 5E).

### 3.4 ATP/DNA measurement

**Fig. 6:**
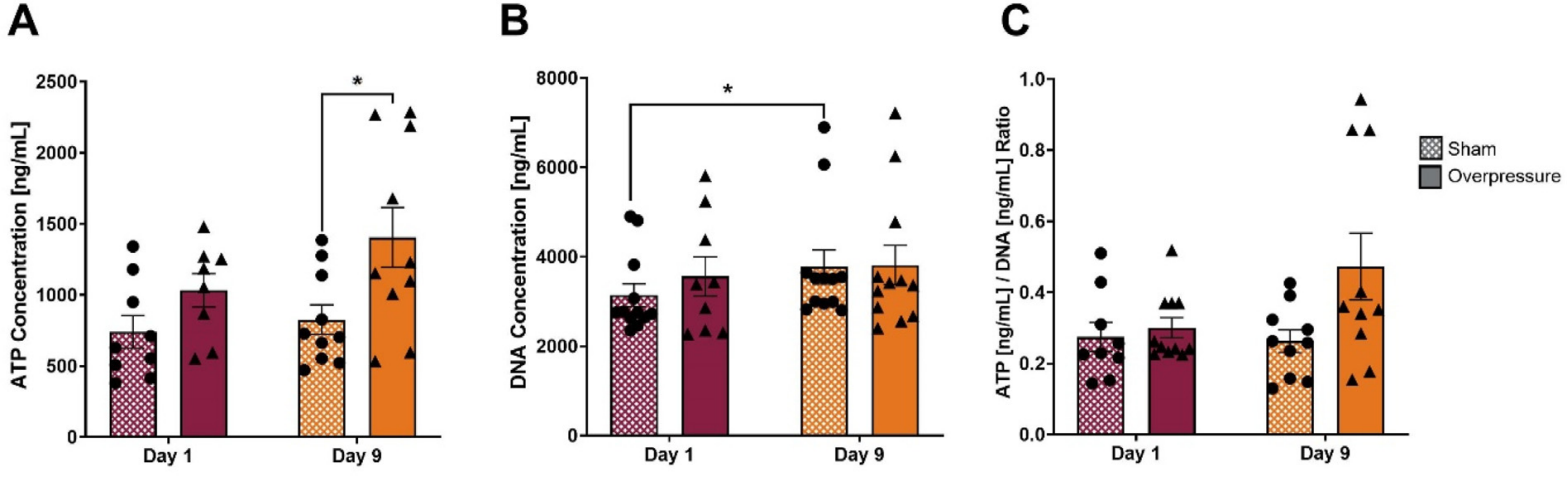
ATP/DNA analysis on OPC encapsulated hydrogels. **(A)** ATP concentration was significantly increased on Day 9 following mechanical insult compared to Day 9 sham. **(B)** DNA concentration of sham over time (Day 1 to Day 9) significantly increased. **(C)** The ATP to DNA concentration ratio was insignificant at all experimental conditions. ∗ Indicates a p-value p ​< ​0.05. The unpaired t-test was used for normally distributed data and Mann–Whitney U test was used for not normally distributed data. Data represented as Mean ​± ​SEM (n ​= ​9–12 hydrogel samples).

The relationship of ATP and DNA concentrations within the OPC lysates was measured (Fig. 6). ATP concentration results showed a significant increase (69.97 ​%, p ​= ​0.0237) in concentration on Day 9 post-overpressure samples compared to the Day 9 sham (Fig. 6A). DNA concentration increased significantly over time for sham group (26.1 ​%, p ​= ​0.0332) when compared from Day 9 sham to Day 1 sham (Fig. 6B). DNA did not increase over time for the post-overpressure group indicating OPC growth and proliferation in the hydrogels had slowed. The ATP to DNA ratio showed a 72.26 ​% increase on Day 9 post-overpressure compared to Day 9 sham; however, the trending result was not statistically significant (p ​= ​0.0753). Comparison between Day 9 post-overpressure and Day 1 post-overpressure also showed no significant change (p ​= ​0.2816) (Fig. 6C).

### 3.5 Gene expression quantification

**Fig. 7:**
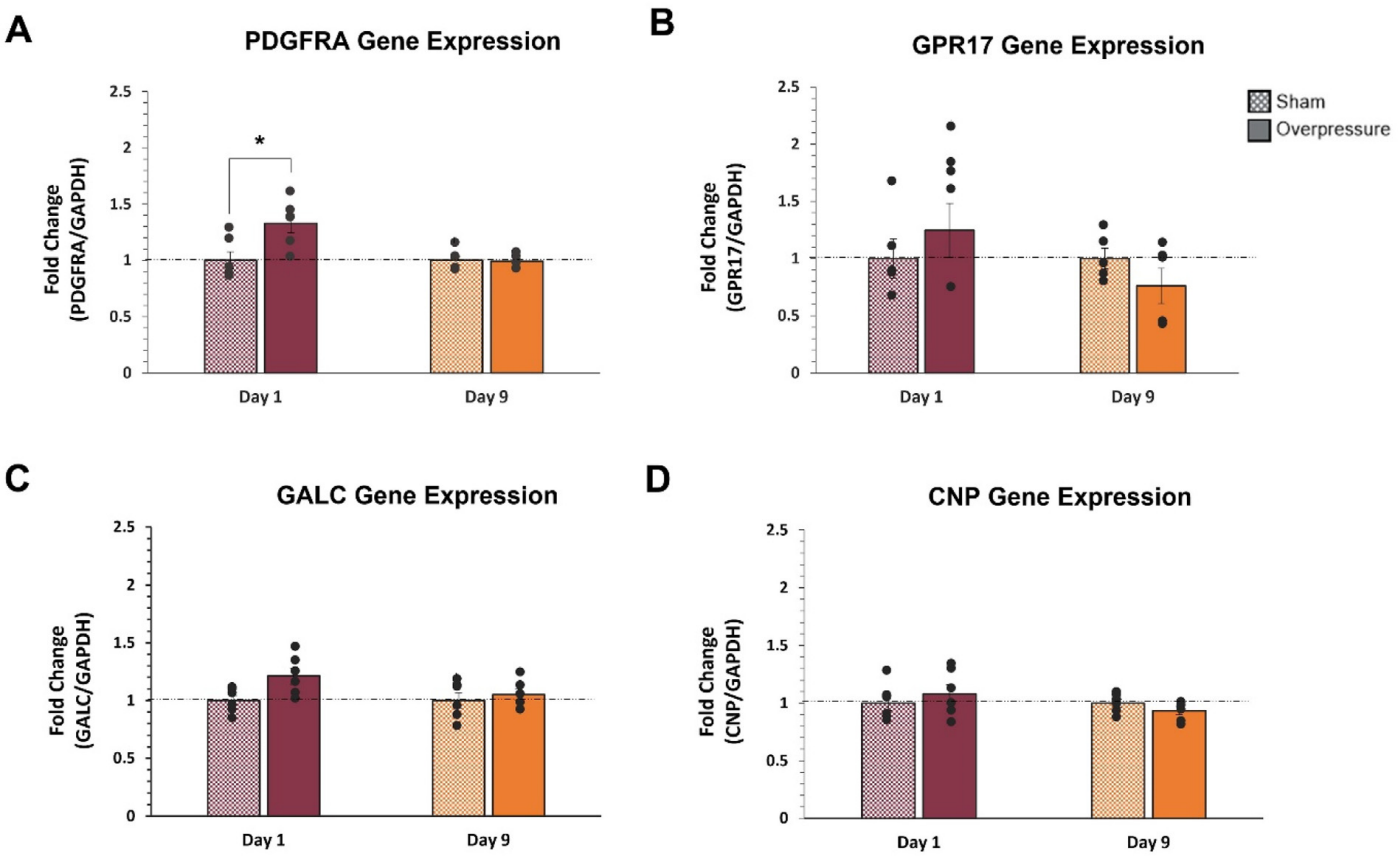
Transcriptomic changes of OPC maturation markers at Day 1 and 9 following mechanical insult. All data was normalized to GAPDH and the time-matched sham. **(A)** A significant increase in expression was observed in *PDGFRA,* suggesting a transcriptomic response in OPCs to the applied overpressure on Day 1. **(B)** The expression of *GPR17* indicated a notable but non-significant increase on Day 1 and an observed decrease on Day 9 following overpressure. **(C)** Expression changes in *GALC* demonstrated a non-significant increase in mRNA on Day 1 in response to the applied overpressure, with it returning to baseline levels at Day 9. **(D)** The expression changes of *CNP* indicated a trending but non-significant decrease from Day 1 to Day 9 following overpressure. ∗ Indicates a p-value <0.05. The unpaired t-test was used for normally distributed data, and the Mann–Whitney U test was used for not normally distributed data. Data are represented as Mean ​± ​SEM (n ​= ​5–6).

Research suggests that OPCs can proliferate and/or differentiate following TBI. qPCR was performed for the maturation markers *PDGFRA*, *GALC*, *GPR17*, and *CNP* to assess the potential transcriptomic changes from the applied mechanical insult in OPCs on Day 1 and Day 9. qPCR analysis revealed significant overexpression in *PDGFRA* (p ​= ​0.012) on Day 1 following insult with a notable decrease in expression observed on Day 9 (Fig. 7A). Interestingly, there were no significant overpressure-specific changes in GPR17, GALC, and CNP expression at either time point. Although the data indicated an increase in *GPR17*, *CNP,* and *GALC* mRNA on Day 1, with *GPR17* and *CNP* mRNA decreasing on Day 9, these results were insignificant.

**Fig. 8:**
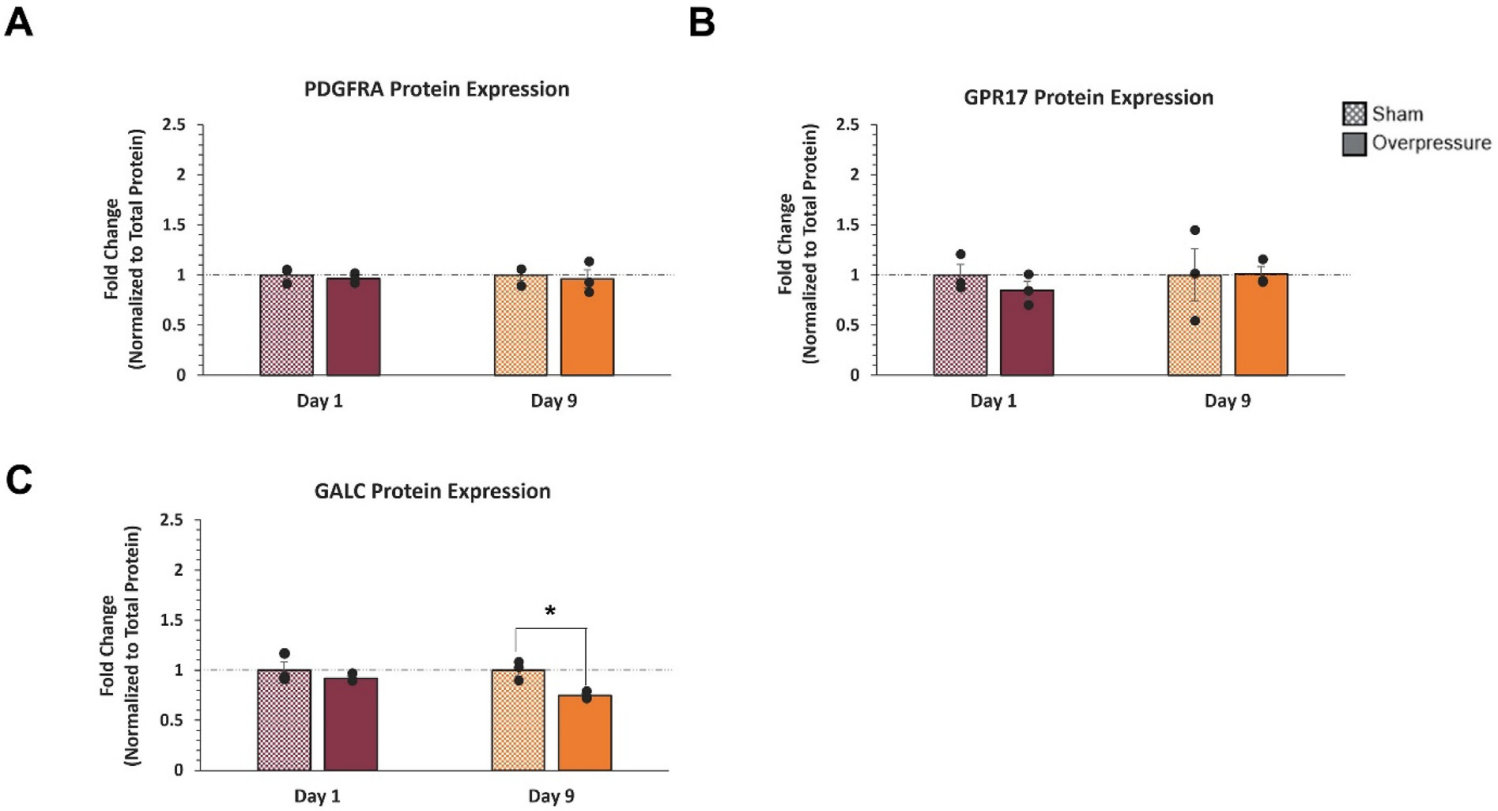
Western Blot analysis of PDGFRA, GALC, and GPR17. All data was normalized to total protein and the respective sham. **(A)** PDGFRA protein expression on Days 1 and 9 following mechanical insult indicated no significant changes. **(B)** GPR17 protein expression showed a non-significant decrease on Day 1, returning to baseline levels on Day 9 following insult. **(C)** GALC protein expression indicated a significant decrease on Day 9 from the applied overpressure. ∗ Indicates a p-value <0.05. The unpaired t-test was used for normally distributed data, and Mann–Whitney U test was used for not normally distributed data. Data represented as Mean ​± ​SEM (n ​= ​3).

**Fig. 9:**
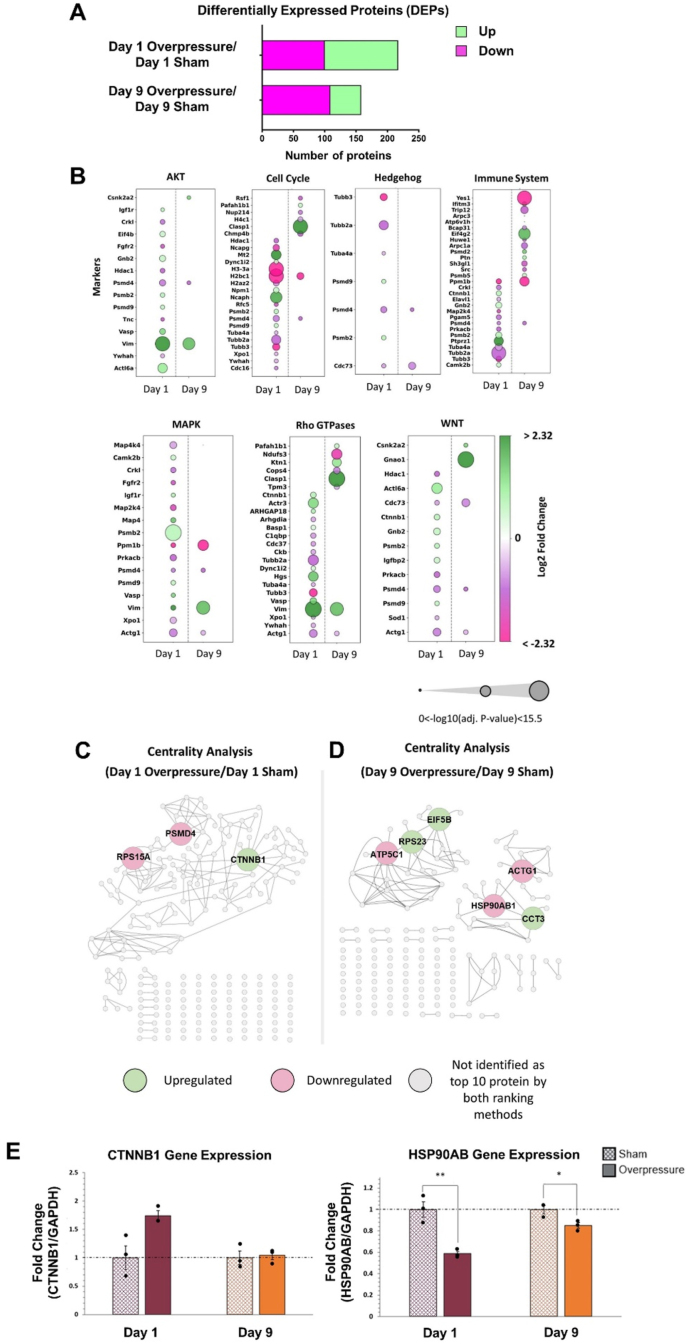
Proteomic analysis on overpressure OPC samples. **(A)** Differentially expressed proteins (DEPs) were identified by the LC-MS/MS experiment and analysis. The overpressure group was normalized to the sham at the same time point. Upregulation (green) and downregulation (pink) of DEPs were color-coded. **(B)** Signaling pathways related to OPC proliferation and differentiation. Proteins were associated with each signaling pathway by the biological interpretation of Cytoscape. Upregulated protein was illustrated in green and downregulated protein in pink. The circle's size significantly changed due to the applied overpressure compared to sham. **(C and D)** Centrality analysis identified the high confidence proteins for Day 1 overpressure normalized to Day 1 sham **(C)** and Day 9 overpressure normalized to Day 9 sham **(D)**. DEPs identified by both ranking methods were color-coded with green and pink. Green represents the upregulation, pink represents the downregulation, and grey represents the proteins not identified by PageRank and weighted rank methods. The PPI generated by Cytoscape 3.10.1 is shown in grey. **(E)** qPCR analysis of *CTNNB1* and *HSP9*0AB. Each experimental condition (sham and overpressure) was normalized to sham of each time point. The dashed line represents the normalized value at 1. ∗ Indicates p ​< ​0.05, ∗∗ Indicates p ​< ​0.01. The unpaired t-test was used for normally distributed data, and the Mann–Whitney U test was used for not normally distributed data. Data represented as Mean ​± ​SEM (n ​= ​3).

### 3.6 Western blot

When assessing changes in protein expression of PDGFRA, GPR17, and GALC through Western blot analysis (Fig. 8), the results showed a significant decrease in GALC (p ​= ​0.013) (Fig. 8C) on Day 9 following overpressure when normalized to the time-matched control. No overpressure-specific changes were observed in the maturation markers PDGFRA and GPR17 at either timepoint.

### 3.7 Proteomic data analysis

To take a broader approach to distinguishing protein changes following insult, the total protein on Days 1 and 9 from OPCs subjected to overpressure was reduced, digested, and subjected to liquid chromatography MS/MS utilizing the protalizer DIA software pipeline. DEPs were identified in Fig. 9A. Functional enrichment and classification of the identified DEPs were performed using the previously described methods. The KEGG and Reactome pathway analysis for Days 1 and 9 suggested DEP involvement in numerous pathways, with literature highlighting their roles in OPC proliferation and differentiation. These pathways included AKT signaling, cell cycle, hedgehog signaling, Mitogen Activated Protein Kinase (MAPK) signaling, Rho GTPases, Wnt signaling, and the immune system (Fig. 9B). In addition to performing functional enrichment analysis, we further analyzed the DEPs using PPI network analysis. As described above, we used Cytoscape 3.10.1 and two ranking algorithms to elucidate DEPs with high confidence in each group. Subsequently, the top 10 proteins obtained from the manual weighting method and those obtained from the PageRank method were mapped to identify high-confidence proteins in each group (Supplemental Tables S1–4). Three high-confidence proteins were found for overpressure Day 1 OPCs, which include catenin beta 1 (CTNNB1), ribosomal protein S15a (RPS15A), and proteasome 26S subunit ubiquitin receptor, non-ATPase 4 (PSMD4) (Fig. 9C). For Day 9 overpressure OPC's, there were 6 high confidence proteins identified including actin gamma 1 (ACTG1), ATP synthase F1 subunit gamma (ATP5F1C), chaperonin containing TCP1 subunit 3 (CCT3), eukaryotic translation initiation factor 5B (EIF5B), heat shock protein 90 alpha family class B member 1 (HSP90AB1), and the ribosomal protein S23 (RPS23) (Fig. 9D).

Two DEPs, CTNNB1 and HSP90AB, were selected for further gene expression analysis based on the result of two ranking methods and their functional relativity to cellular response following external stress (Fig. 9E).^59^, ^60^, ^61^, ^62^, ^63^ qPCR analysis demonstrated significant post-overpressure downregulations in *HSP9*0AB on Day 1 (p ​= ​0.002) and Day 9 (p ​= ​0.032) (Fig. 9E). *CTNNB1* gene expression was increased following applied overpressure on Day 1. However, it did not show a statistically significant change (p ​= ​0.054).

## 4 Discussion and conclusion

In this study, we examined the influence of mechanical insult on the cellular and molecular responses of OPCs, focusing on proliferation- and maturation-related molecules. OPCs cultured in the NorHA hydrogel *in vitro* platform were subjected to an overpressure injury model. An applied mechanical insult with average peak overpressures of 19.08 ​± ​6.53 psi and characteristic wave properties, replicating conditions of a mild TBI.^54^^,^^55^^,^^64^

We conducted rheology testing to assess the mechanical properties of NorHA following high-rate mechanical insult. The mechanical properties of the NorHA hydrogels followed a pattern where higher NorHA concentrations resulted in more crosslinks and a stiffer gel (Fig. 4).^45^ Gels exposed to mechanical insult stiffened compared to sham gels at all three NorHA concentrations, suggesting that the gel matrix was slightly compacted by overpressure exposure (Fig. 4C). When encapsulating OPCs, hydrogel stiffness decreased at all three NorHA concentrations (Fig. 4B). This result was also consistent with previous findings which showed that encapsulating cells reduced the stiffness of covalently crosslinked, elastic hydrogels, as cells effectively served as defects in the polymer network that had a mesh size orders of magnitude smaller than the diameter of a typical cell.^45^ In addition, Feng et al. measured the elastic modulus of mouse brain tissue following a controlled cortical impact test. They observed minimal changes in tissue elastic property on different brain locations at 12 ​h following injury.^65^ In cell-containing hydrogels, the 2 ​% NorHA samples exposed to applied mechanical insult were stiffer than the corresponding sham samples, indicating compaction of the gels following overpressure exposure (Fig. 4D). However, in more compliant hydrogel formulations (1 and 1.5 ​% NorHA), the samples post-insult were less stiff than the corresponding sham samples. This may indicate that, at lower crosslinking densities, exposure to the mechanical loading leads to crosslink cleavage rather than compaction. Notably, these results were specific to cell-containing gels and were not observed in acellular hydrogel samples. Because cells were present in the precursor solution prior to gelation, crosslinks in the hydrogel system were formed around the cells. The cell presence contributes to crosslinking disruptions the network which has a mesh size orders of magnitude smaller than a cell. As such, cell-containing samples were more susceptible to damage resulting from the mechanical loading. Previous studies have used nanoindentation or atomic force microscopy (AFM) to determine local material properties in NorHA gels.^47^^,^^66^ Future work could implement these techniques to determine the impacts of mechanism loading or presence of cells/clusters to hydrogel structural properties on a local level. For this work, we assume that any changes in hydrogel stiffness following the insult were minimal and unlikely to affect the cellular and molecular responses of OPCs. Therefore, 1.5 ​% NorHA hydrogel was chosen as a controlled material, allowing us to focus on the effects of the high-rate mechanical insult rather than variations in hydrogel mechanical properties.

Hlavac et al. conducted 3D culture experiments on astrocytes and found high viability following high-rate overpressure.^49^ Our results demonstrated that OPC microtissues post-overpressure exhibited high viability (90.00 ​± ​2.09 ​% on Day 1 and 93.00 ​± ​2.09 ​% on Day 9), similar to the viability for sham microtissues (92.08 ​± ​1.64 ​% on Day 1 and 91.44 ​± ​2.37 ​% on Day 9) (Fig. 5A). Similar to the findings of Hlavac et al., we observed that the applied overpressure did not result in significant OPC death up to 9 days post-exposure. The necrotic core of the spheroid is often an issue of spheroid culture;^67^ however, this study demonstrated that following two weeks of cell culture, spheroid formation within a hydrogel did not generate a necrotic core (Supplemental Fig. S1). This result indicated that the NorHA hydrogel provided sufficient diffusion of nutrients for 14 days of the study.

To assess the proliferation of OPCs following high-rate mechanical insult, we analyzed changes in spheroid volume and DNA concentration. Spheroid analysis of live/dead images showed no significant growth following overpressure in total spheroid volume and mean spheroid volume within the hydrogel (Fig. 5C and D). In addition to spheroid analysis, cell proliferation was also quantified by measuring the DNA concentration of cells in a whole hydrogel (Fig. 6B). The result supported no significant proliferation following the insult, except for the significant increase over time (Day 9 sham compared to the Day 1 sham, p ​= ​0.0332). These findings suggest that the OPCs in this 3D system may be in a quiescent state or beginning to undergo differentiation.^68^^,^^69^ Since the DNA analysis is more comprehensive than the spheroid imaging analysis with limited ROIs, there may have been a continuation of cell proliferation within the sham samples at a slower rate and inhibited cell growth on the overpressure samples (Fig. 6B). The drawback of this result is that there is a possibility of capacity limitation for the cells to grow within the hydrogel matrix since there was no significant difference in the spheroid volume to hydrogel volume ratio (Fig. 5E). Therefore, future studies targeting specific proliferation marker such as bromodeoxyuridine (BrdU) or 5-ethynyl 2′-deoxyuridine (EdU) to quantify cells actively synthesizing DNA would expand the insight needed to confirm the result of changes in DNA concentration.^66^

Previous *in vitro* TBI models demonstrated that overpressure exposure caused astrocyte reactivity and led to metabolic activity aberrations within 48 ​h after injury.^48^^,^^49^^,^^70^ These findings are consistent with the results obtained in our metabolic activity assay. Our *in vitro* model showed a significant increase in ATP concentration due to applied overpressure on Day 9 (p ​= ​0.0237), which indicated that the applied mechanical insult influences cell metabolic activity (Fig. 6C). Although our results showed a significant change only on Day 9, potential activation of mechanotransduction signaling after applied mechanical insult may have caused changes in ATP synthesis and consumption.^49^^,^^71^, ^72^, ^73^, ^74^ Although ATP dysregulation was observed, the ratio between ATP and DNA showed no significant difference. This result suggests that the ATP concentration per cell population within a hydrogel did not significantly change after mechanical insult. Future studies are needed to confirm the mode of these metabolic dysregulations.

Previous work with astrocytes suggests that the overpressure loading might trigger the initiation of mechanotransduction molecules (FAK, integrin, and vinculin) as well as astrocyte reactivity as indicated by an increase in GFAP expression.^49^ Recent studies indicate that a cell's rapid force sensing capability and subsequent accumulation of mechanotransduction molecules (pFAK and pMyosin IIa) can significantly upregulate the yes-associated protein (YAP)/the transcription coactivator with PDZ-binding motif (TAZ) activation.^75^ YAP/TAZ signaling activation has been associated with regulation of OPC maturation process.^76^ For OPC-specific responses, proliferation at an early time point following injury has been reported in various preclinical TBI models.^10^^,^^11^^,^^77^ Our study showed that the expression of the cell proliferation marker *PDGFRA* was significantly upregulated at Day 1 following insult (p ​= ​0.012), indicating a preference towards OPC self-renewal (Fig. 7A). This result indicates transcriptomic dysregulations in *PDGFRA* following insult, which aligns with other models. However, no significant proteomic changes in PDGFRA were observed, suggesting that the applied mechanical insult was not significant enough to alter the cell function of proliferation (Fig. 8A).

The GALC protein significantly decreased at Day 9 (p ​= ​0.013) (Fig. 8C) and is most notable for its association with myelin development, as it is more enriched in OLs than OPCs.^78^, ^79^, ^80^ This result may suggest that the mechanical insult causes a sustained inhibition of OPC maturation. No transcriptomic or proteomic dysregulations in CNP were observed. This result is consistent with the lack of CNP expression in the MADM OPC immortalized cell line. Thus, CNP was excluded from the Western blot analysis. Future studies with primary cells or stem cell-derived OPCs are necessary to confirm the effect of applied mechanical insult on the OPC to OL maturation process.

Correspondence between gene and protein expressions varies depending on dynamic cellular processes. While gene expression of *PDGFRA*, *GPR17*, and *GALC* increased on Day 1 following applied overpressure, protein expressions did not show any changes at the same time point. Only GALC protein expression significantly decreased on Day 9 following the insult. However, its gene expression did not change. It may be necessary to assess time-dependent effects between Day 1 and Day 9 to confirm the effect of mechanical insult on *PDGFRA* gene expression and GALC protein expression.

In addition to the effect of applied overpressure on OPC to OL maturation markers, proteomic analysis was conducted on Days 1 and 9 following overpressure. This study focused on identifying dysregulated proteins specific to OPC proliferation and differentiation (Fig. 9). Proteomic analysis on the Day 1 group identified CTNNB1 as a central protein within the network. Numerous studies highlight its importance as a regulatory factor of OL development through canonical WNT signaling (Fig. 9B and C).^59^, ^60^, ^61^ Similarly, the deletion of beta-catenin in OPCs in a rodent model reduced proliferation, suggesting the importance of this signaling pathway for tissue regeneration after spinal cord injury.^81^ This finding may imply that the high-rate pressure wave propagation causes activation of the WNT signaling by overexpressing CTNNB1 to induce the recovery response of OPCs. Consequently, the other two influential proteins identified through network analysis (RPS15A and PSMD4) have been widely understudied in the context of mechano-stimulation, which presents an exciting opportunity for further investigation. To further elucidate the molecular response of CTNNB1, we quantified the gene expression of *CTNNB1* following the mechanical insult to understand the effect of overpressure at the mRNA level (Fig. 9E). While no significant changes were observed on Day 9, increased expression was detected on Day 1 following the mechanical insult, consistent with the findings from the proteomic analysis. This finding implies that the CTNNB1 can be a potential mechanosensing target in OPCs that responds to a mechanical insult, and further analysis is necessary to understand how signaling transduction involving CTNNB1 affects OPC proliferation and maturation.

When performing proteomic analysis on the Day 9 group, HSP90AB1 was identified to be one of the most influential proteins within the network (Fig. 9D). HSP90AB1 is a molecular chaperone relating to the regulation of protein folding in response to external stress. Interestingly, the role of HSP90AB1 also expands to cell cycle control and signal transduction. Multiple studies have reported that the anti-HSP90beta antibody was observed in cerebrospinal fluid (CSF) in multiple sclerosis (MS) patients, having the ability to bind to the OPC surface and, in some cases, lead to OPC death.^62^^,^^63^ In addition to the proteomic analysis, the gene expression of *HSP9*0AB was measured to better understand its involvement. We observed significant decreases on Day 1 (p ​= ​0.002) and Day 9 (p ​= ​0.032) following mechanical insult (Fig. 9E). This is consistent with the proteomic analysis; however, at the protein level, the expression was downregulated only at Day 9. *HSP9*0AB downregulation following mechanical insult may indicate the cell attempt to promote cell survival following the external insult.^82^ Further investigation is required to understand the molecular effects of HSP90AB dysregulation on OPCs and its correlation to cell proliferation and maturation following applied mechanical insult.

Proteomic analysis revealed other potential molecular targets (ACTG1, ATP5F1C, RPS23, EIF5B, CCT3). ACTG1 may be particularly interesting as an ACTG1 variant in post-mortem human brain tissue is associated with the loss of oligodendrocytes.^83^ However, its correlation to OPC maturation or differentiation needs further investigation. ATP5F1C, RPS23, EIF5B, and CCT3 are understudied in the context of OPC injury responses, warranting future investigations to elucidate their roles in the injury response following applied mechanical insult.

This study characterized the impact of an applied mechanical insult on OPC by demonstrating the cellular and molecular response in the biofidelic NorHA hydrogel 3D cell culture system. Although there are some limitations, including the use of an immortalized cell line (p53 knockout) and a limited time course, future studies can leverage this cell culture platform to further investigate by conducting assays at additional time points and quantifying molecular targets related to the identified signaling pathways. While our model system was not identical to native brain tissue, we have evidence suggesting that it does resemble native conditions in various imperative ways, including ECM deposition. Additionally, the tunability of our system allows us to close this gap as we move forward with this work. For example, while cell shape was not representative of native tissue in the current system, evidence shows that the introduction of electrospun fibers produce cell shapes indicative of mature OPC.^66^ Modifying our model system to incorporate fibers could help improve relevance of our model in future experiments. As such, further studies are vital to translate the proliferation and maturation of OPCs for advanced regenerative medicine therapies.

## CRediT authorship contribution statement

**Ryosuke Yokosawa:** Writing – review & editing, Writing – original draft, Visualization, Validation, Software, Methodology, Investigation, Formal analysis, Data curation, Conceptualization. **Rachel A. Mazur:** Writing – review & editing, Writing – original draft, Visualization, Methodology, Investigation, Formal analysis, Data curation, Conceptualization. **Kelsey A. Wilson:** Writing – review & editing, Writing – original draft, Visualization, Software, Methodology, Investigation, Formal analysis, Data curation, Conceptualization. **Jacob H. Lee:** Writing – review & editing, Methodology, Formal analysis, Data curation. **Noah W. Showalter:** Writing – review & editing, Software, Methodology, Data curation. **Kyle J. Lampe:** Writing – review & editing, Writing – original draft, Visualization, Validation, Supervision, Software, Resources, Project administration, Methodology, Investigation, Funding acquisition, Formal analysis, Data curation, Conceptualization. **Pamela J. VandeVord:** Writing – review & editing, Writing – original draft, Visualization, Validation, Supervision, Software, Resources, Project administration, Methodology, Investigation, Funding acquisition, Formal analysis, Data curation, Conceptualization.

## Ethical approval

This study does not contain studies with animal or human subjects performed by any of the authors.

## Declaration of competing interest

All authors have no competing interests to declare.
