# Supplementary material for: High-rate mechano-stimulation alters proliferation- and maturation-related signaling of oligodendrocyte precursor cells in a 3D hydrogel
Source: Mechanobiol Med. 2025 Mar 11;3(3):100126. doi: 10.1016/j.mbm.2025.100126 (PMC12163403; doi:10.1016/j.mbm.2025.100126)
Supplement: Multimedia component 1 [file mmc1.docx]

**Appendix A. Supplementary data**

| **Supplemental Table S1.** Top 10 DEPs identified by the centrality analysis using PageRank method for Day 1 overpressure normalized to Day 1 sham group. | | |
| --- | --- | --- |
| ***Protein*** | ***Score*** | ***Description from GeneCards**** |
| ***CTNNB1*** | 0.0264 | Key downstream component of the canonical Wnt signaling pathway. |
| ***ACTG1*** | 0.0203 | Actin gamma 1, encoded by this gene, is a cytoplasmic actin found in all cell types. |
| ***HDAC1*** | 0.0147 | Histone acetylation and deacetylation, catalyzed by multi-subunit complexes, play a key role in the regulation of eukaryotic gene expression. |
| ***DNAJB11*** | 0.0144 | This gene encodes a soluble glycoprotein of the endoplasmic reticulum (ER) lumen that functions as a co-chaperone of binding immunoglobulin protein, a 70 kilodalton heat shock protein chaperone required for the proper folding and assembly of proteins in the ER. |
| ***H3F3B*** | 0.0142 | Constitutes the predominant form of histone H3 in non-dividing cells and is incorporated into chromatin independently of DNA synthesis. |
| ***RPS15A*** | 0.0142 | Structural component of the ribosome. Required for proper erythropoiesis; Belongs to the universal ribosomal protein uS8 family. |
| ***PRKACB*** | 0.0137 | A catalytic subunit of cAMP (cyclic AMP)-dependent protein kinase, which mediates signaling though cAMP. |
| ***SNRPD1*** | 0.0130 | Plays a role in pre-mRNA splicing as a core component of the spliceosomal U1, U2, U4 and U5 small nuclear ribonucleoproteins (snRNPs), the building blocks of the spliceosome. |
| ***PSMD4*** | 0.0126 | Component of the 26S proteasome, a multiprotein complex involved in the ATP-dependent degradation of ubiquitinated proteins. |
| ***ELAVL1*** | 0.0125 | RNA-binding protein that binds to the 3'-UTR region of mRNAs and increases their stability. |

*GeneCards website (<https://www.genecards.org/>)

| **Supplemental Table S2.** Top 10 DEPs identified by the centrality analysis using weighted rank method for Day 1 overpressure normalized to Day 1 sham group. | | | | | | |
| --- | --- | --- | --- | --- | --- | --- |
| ***Protein*** | ***Betweenness*** | ***Closeness*** | ***Degree*** | ***Eccentricity*** | ***Stress*** | ***Weighted Sum*** |
| ***CTNNB1*** | 0.479928 | 0.194030 | 11 | 16 | 13140 | 5261.08 |
| ***RPS15A*** | 0.397000 | 0.179620 | 8 | 11 | 12280 | 4915.66 |
| ***EIF3F*** | 0.373537 | 0.165342 | 4 | 11 | 11608 | 4645.65 |
| ***PSMD4*** | 0.363191 | 0.152047 | 7 | 12 | 11398 | 4562.64 |
| ***SOD1*** | 0.265683 | 0.138482 | 6 | 13 | 9994 | 4000.81 |
| ***PARK7*** | 0.235250 | 0.125908 | 2 | 14 | 9472 | 3790.90 |
| ***SDHB*** | 0.223301 | 0.115172 | 5 | 15 | 9302 | 3723.89 |
| ***POLR2E*** | 0.333682 | 0.201161 | 3 | 14 | 8204 | 3284.04 |
| ***BRIX1*** | 0.306168 | 0.195857 | 4 | 13 | 7728 | 3093.83 |

| **Supplemental Table S2 continued.** |
| --- |
| ***Description from GeneCards**** |
| Key downstream component of the canonical Wnt signaling pathway. |
| Structural component of the ribosome. Required for proper erythropoiesis; Belongs to the universal ribosomal protein uS8 family. |
| Component of the eukaryotic translation initiation factor 3 (eIF-3) complex, which is required for several steps in the initiation of protein synthesis. |
| Component of the 26S proteasome, a multiprotein complex involved in the ATP-dependent degradation of ubiquitinated proteins. |
| SOD1 binds copper and zinc ions and is one of two isozymes responsible for destroying free superoxide radicals in the body. |
| Multifunctional protein with controversial molecular function which plays an important role in cell protection against oxidative stress and cell death acting as oxidative stress sensor and redox-sensitive chaperone and protease. |
| The iron-sulfur protein subunit of the succinate dehydrogenase (SDH) enzyme complex which plays a critical role in mitochondria. |
| DNA-dependent RNA polymerase catalyzes the transcription of DNA into RNA using the four ribonucleoside triphosphates as substrates. |
| Enables RNA binding activity. |

*GeneCards website (<https://www.genecards.org/>)

| **Supplemental Table S3.** Top 10 DEPs identified by the centrality analysis using weighted rank method for Day 9 overpressure normalized to Day 9 sham group. | | |
| --- | --- | --- |
| ***Protein*** | ***Score*** | ***Description from GeneCards**** |
| ***ACTG1*** | 0.0315 | Actin gamma 1, encoded by this gene, is a cytoplasmic actin found in all cell types. |
| ***ATP5F1C***  ***(ATP5C1)*** | 0.0252 | Mitochondrial membrane ATP synthase (F(1)F(0) ATP synthase or Complex V) produces ATP from ADP in the presence of a proton gradient across the membrane which is generated by electron transport complexes of the respiratory chain. |
| ***HSP90AB1*** | 0.0242 | Molecular chaperone that promotes the maturation, structural maintenance and proper regulation of specific target proteins involved for instance in cell cycle control and signal transduction. |
| ***H2BC1*** | 0.0221 | Variant histone specifically required to direct the transformation of dissociating nucleosomes to protamine in male germ cells (By similarity). |
| ***RPS23*** | 0.0217 | Component of the ribosome, a large ribonucleoprotein complex responsible for the synthesis of proteins in the cell. |
| ***EIF5B*** | 0.0208 | Ribosome-dependent GTPase that promotes the joining of the 60S ribosomal subunit to the pre-initiation complex to form the 80S initiation complex with the initiator methionine-tRNA in the P-site base paired to the start codon. |
| ***CCT3*** | 0.0188 | Component of the chaperonin-containing T-complex (TRiC), a molecular chaperone complex that assists the folding of proteins upon ATP hydrolysis. |
| ***NDUFS3*** | 0.0172 | Core subunit of the mitochondrial membrane respiratory chain NADH dehydrogenase (Complex I) which catalyzes electron transfer from NADH through the respiratory chain, using ubiquinone as an electron acceptor. |
| ***ALB*** | 0.0168 | The regulation of blood plasma colloid osmotic pressure and acts as a carrier protein for a wide range of endogenous molecules including hormones, fatty acids, and metabolites, as well as exogenous drugs. |
| ***PLRG1*** | 0.0168 | Involved in pre-mRNA splicing as component of the spliceosome. |

*GeneCards website (<https://www.genecards.org/>)

| **Supplemental Table S4.** Top 10 DEPs identified by the centrality analysis using weighted rank method for Day 9 overpressure normalized to Day 9 sham group. | | | | | | |
| --- | --- | --- | --- | --- | --- | --- |
| ***Protein*** | ***Betweenness*** | ***Closeness*** | ***Degree*** | ***Eccentricity*** | ***Stress*** | ***Weighted Sum*** |
| ***Hsp90ab1*** | 0.649758 | 0.489796 | 6 | 4 | 420 | 170.49 |
| ***Actg1*** | 0.663043 | 0.480000 | 8 | 4 | 416 | 169.49 |
| ***ATP5F1C (Atp5c1)*** | 0.486495 | 0.547619 | 11 | 4 | 376 | 154.36 |
| ***Rps23*** | 0.443347 | 0.589744 | 8 | 3 | 334 | 136.55 |
| ***Eif5b*** | 0.250329 | 0.469388 | 7 | 3 | 206 | 84.97 |
| ***Cct3*** | 0.251208 | 0.387097 | 5 | 5 | 190 | 78.15 |
| ***Cops4*** | 0.161836 | 0.375000 | 3 | 5 | 112 | 46.32 |
| ***Capzb*** | 0.159420 | 0.352941 | 3 | 5 | 104 | 43.12 |
| ***Mrpl12*** | 0.095520 | 0.500000 | 4 | 3 | 100 | 41.63 |
| ***Rpl18a*** | 0.120553 | 0.450980 | 6 | 3 | 94 | 39.83 |

| **Supplemental Table S4 continued.** |
| --- |
| ***Description from GeneCards**** |
| Molecular chaperone that promotes the maturation, structural maintenance and proper regulation of specific target proteins involved for instance in cell cycle control and signal transduction. |
| Actin gamma 1, encoded by this gene, is a cytoplasmic actin found in all cell types. |
| Mitochondrial membrane ATP synthase (F(1)F(0) ATP synthase or Complex V) produces ATP from ADP in the presence of a proton gradient across the membrane which is generated by electron transport complexes of the respiratory chain. |
| Component of the ribosome, a large ribonucleoprotein complex responsible for the synthesis of proteins in the cell. |
| Ribosome-dependent GTPase that promotes the joining of the 60S ribosomal subunit to the pre-initiation complex to form the 80S initiation complex with the initiator methionine-tRNA in the P-site base paired to the start codon. |
| Component of the chaperonin-containing T-complex (TRiC), a molecular chaperone complex that assists the folding of proteins upon ATP hydrolysis. |
| Component of the COP9 signalosome complex (CSN), a complex involved in various cellular and developmental processes. |
| F-actin-capping proteins bind in a Ca(2+)-independent manner to the fast growing ends of actin filaments (barbed end) thereby blocking the exchange of subunits at these ends. |
| As a component of the mitochondrial large ribosomal subunit, it plays a role in mitochondrial translation. |
| Component of the large ribosomal subunit. The ribosome is a large ribonucleoprotein complex responsible for the synthesis of proteins in the cell. |

*GeneCards website (<https://www.genecards.org/>)

| 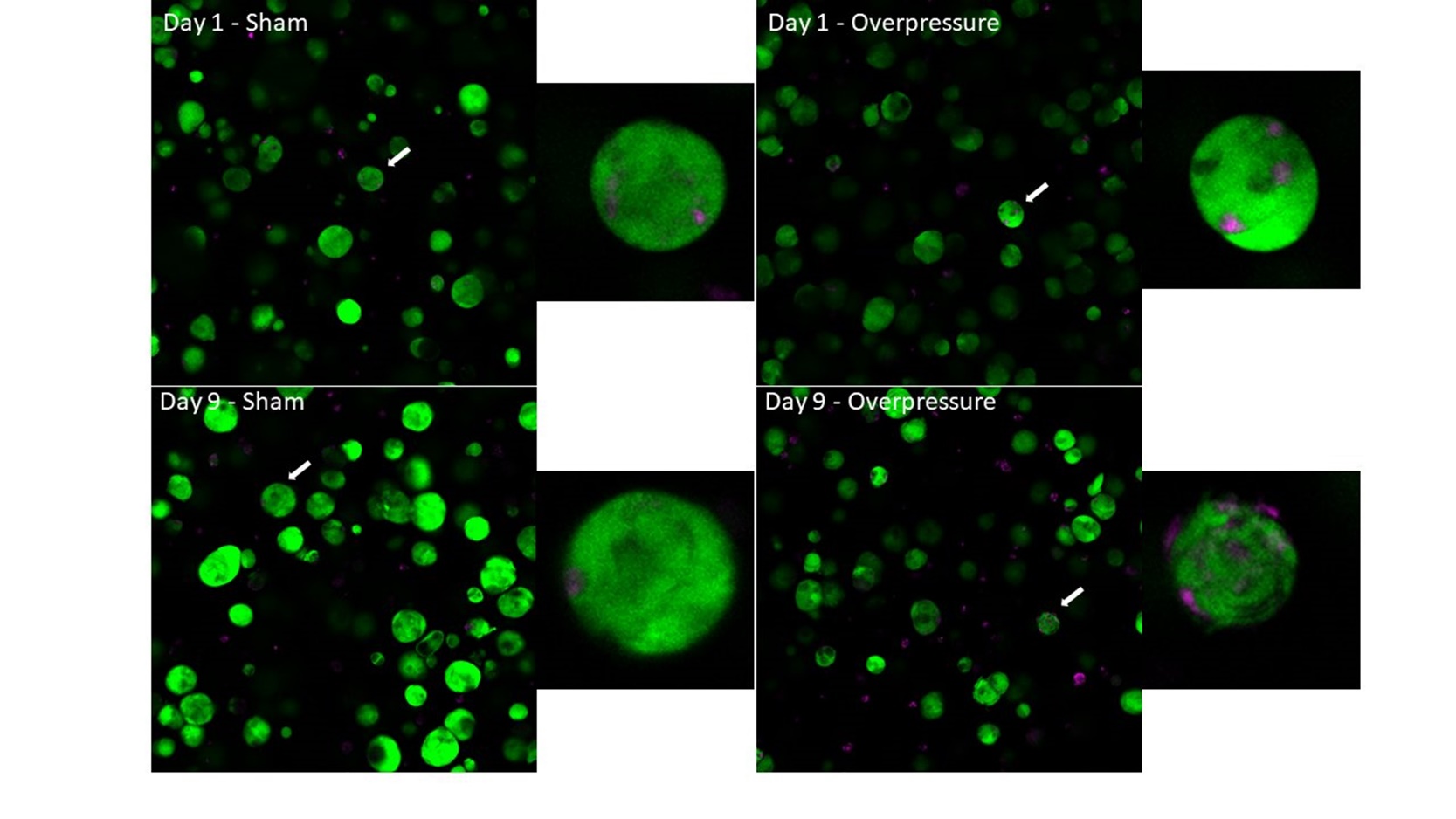 |
| --- |
| **Supplemental Figure S1.** Representative images of spheroid middle section to show cell condition within a spheroid. These images show no necrotic cores inside the spheroid rather individual dead cell is sporadically attached to the spheroid. White arrow indicates the location of magnified spheroid. Magenta = dead cell, Green = live cell. |
